# Pharmacokinetic and bioequivalence evaluation of two abiraterone acetate tablets under fasting conditions in healthy Chinese volunteers

**DOI:** 10.1016/j.clinsp.2026.101077

**Published:** 2026-08-11

**Authors:** Jinjin Shi, Xiaoshan Zhu, Xu Zuo, Ancheng Kuang, Yang Wang, Cheng Liu, Guiying Chen, Tiandong Zhang

**Affiliations:** a Phase I Clinical Trial Research Laboratory, Xinxiang Central Hospital, The Fourth Clinical College of Xinxiang Medical University, Xinxiang, China; b Wuhan Jiulong Humanwell Pharmaceutical Co., Ltd., Wuhan, China; c Gao An People’s Hospital, Gao An, China; d Wuhan Hongren Biopharmaceutical Inc., Wuhan, China

**Keywords:** Bioequivalence test, Abiraterone acetate, Pharmacokinetics, Safety

## Abstract

**Objectives:**

The combination of abiraterone acetate and prednisone is administered to patients with metastatic castration-resistant prostate cancer who have not responded to docetaxel-based chemotherapy. This study was conducted to assess the bioequivalence between generic and branded 250 mg abiraterone acetate tablets in healthy Chinese participants under fasting conditions.

**Method:**

A single-dose, randomized, open-label, three-way, three-period, partially replicated crossover trial design was developed. A total of 45 healthy participants were enrolled and randomly assigned to three groups. Each participant received a single oral dose of either the test formulation (generic abiraterone acetate) or the reference formulation (brand-name abiraterone acetate), followed by a 7-day washout period before receiving the alternate formulation in subsequent periods. Blood samples were collected before dosing and up to 72 h post-dose to determine plasma abiraterone concentrations. These data were used to generate concentration-time curves and to calculate key pharmacokinetic parameters.

**Results:**

Our results indicated that there were no notable differences between the two formulations in terms of peak plasma concentration (C__max_), area under the curve from time zero to the last quantifiable concentration (AUC_0-t_), and area under the curve from time zero to infinity (AUC_0-∞_).

**Conclusions:**

Statistical evaluation confirmed that the generic and branded formulations were bioequivalent when administered under fasting conditions.

## Highlights

• C__max_ shows no significant difference between the test and reference formulations.
• Generic and branded abiraterone show comparable absorption in healthy subjects.
• The test formulation is safe and may be an alternative for Chinese patients.

## Introduction

Prostate Cancer (PCa) is among the most prevalent malignancies of the male genitourinary tract. Its occurrence is especially frequent in Europe and North America, where it stands as the second most common cause of cancer-related mortality in men, surpassed only by lung cancer.^1^

Data from 2019^2^ show that approximately 174,650 new diagnoses and 31,620 deaths due to prostate cancer were reported in the United States that year. In China, the incidence rate has been progressively increasing, leading to a growing burden on the healthcare system.^3^ For early-stage prostate cancer, surgical intervention remains the primary treatment option.^4^ In contrast, for intermediate to advanced stages, particularly when distant metastases are present, treatment typically involves systemic therapies, with endocrine (hormonal) therapy playing a central role.^5^^,^^6^ Endocrine therapy can be broadly divided into two categories based on their mechanisms and therapeutic targets: castration therapy and anti-androgen therapy. Castration therapy acts on the hypothalamic-pituitary-gonadal axis, either by surgically removing or pharmacologically suppressing testicular testosterone production. This can be achieved through surgical castration (orchiectomy) or the administration of luteinizing hormone-releasing hormone analogues. In contrast, anti-androgen therapy functions by competitively inhibiting the binding of androgens to androgen receptors in prostate cells, thereby blocking their biological activity at the target tissue level, such as the prostate or adrenal glands.^7^^,^^8^

Abiraterone acetate is a prodrug of abiraterone, a selective and irreversible inhibitor of the 17α-hydroxylase/C17,20-lyase (CYP17) enzyme. This enzyme is expressed in both testicular and adrenal tissues and catalyzes the conversion of norethisterone and progesterone into testosterone precursors, specifically dehydroepiandrosterone, and androstenedione through 17α-hydroxylation and C17,20 bond cleavage.^9^ By inhibiting CYP17, abiraterone effectively blocks testosterone synthesis in the testes, adrenal glands, and prostate tumors. Since prostate cancer is an androgen-dependent malignancy, suppression of testosterone production is a key pharmacological strategy for controlling disease progression.

Abiraterone acetate, developed by Cougar Biotechnology, Inc. (acquired by Johnson & Johnson on July 9, 2009), was approved by the U.S. Food and Drug Administration (FDA) for marketing on April 28, 2011.^10^^,^^11^ As a novel androgen biosynthesis inhibitor, abiraterone works by blocking multiple steps in androgen production, including inhibition of 17α-hydroxylase, C17,20-lyase, and associated enzymes, in the adrenal glands, testes, and tumors. When combined with prednisone, it can also help alleviate symptoms associated with secondary hypercortisolism. According to the Opinions of the General Office of the State Council on the Consistency Evaluation of Quality and Efficacy of Generic Drugs (2016), issued by the National Medical Products Administration (NMPA), Bioequivalence (BE) studies are required to ensure the therapeutic equivalence between generic drugs and their brand-name counterparts. Based on the Directory of Reference Formulations^12^ (Batch 8, https://www.nmpa.gov.cn/xxgk/ggtg/ypggtg/ypqtggtg/20170721092001100.html), The reference formulation used in this trial was abiraterone acetate tablets manufactured by Janssen-Cilag International N.V. In the study, blood samples were obtained from healthy volunteers after oral administration of both the test (generic) and reference (brand-name) formulations under fasting conditions. Plasma concentrations of the drug were determined using a validated Liquid Chromatography – Mass Spectrometry (LC-MS) technique. Pharmacokinetic (PK) parameters were calculated and used to assess bioequivalence of the two formulations.

## Subjects and materials

### Testing drugs

The generic version of abiraterone acetate (or the reference formulation, T) (250 mg, batch number 61,220,702, content purity 96.9%, expiration date July 6, 2024) was manufactured by Wuhan Jiulong Renfu Pharmaceutical Co., Ltd. The original formulation of abiraterone acetate tablets (or the reference formulation, R) (250 mg, batch number CHSGB, content purity 98.93%, and expiration date May 2023) was sourced from Patheon, Inc. Production was carried out according to the guidelines issued by the NMPA for reference preparation.^12^ The drug was stored at 15°–30 °C.

### Research design

This clinical trial was carried out in the Phase I Unit of Xinxiang Central Hospital, with ethical approval granted by the hospital’s Ethics Committee (approval numbers: 2022–056 and 2022–541). The trial was registered on the Chemical Drug Bioequivalence Trial Information Platform (www.chinadrugtrials.org.cn; 09/11/2022; CTR20222731). All procedures strictly complied with the Declaration of Helsinki, as well as relevant national and local regulations. Written informed consent was obtained from all participants prior to study initiation.

Based on the Technical Guidelines for the Bioequivalence Study of Abiraterone Acetate Tablets,^13^ healthy male volunteers were enrolled in this trial. Previous studies^14^ report that the interindividual variability for fasting oral administration of abiraterone acetate tablets exceeds 30%. Therefore, this study assumed high variability, with an intra-subject Coefficient of Variation (intra-CV) of 45%. The expected geometric mean ratio for the primary pharmacokinetic parameter between the test and reference formulations was set at 90%, with a significance level (α) of 0.05. The bioequivalence acceptance range was calculated using e(ln(0.80)/0.25 * SWR)–e(ln(0.80)/0.25 * SWR), where SWR represents the within-subject ratio, and ranged from 72.15% to 138.59%. Using PASS 11 software, with 80% statistical power, a 20% dropout rate, and a three-period crossover design, it was determined that 45 participants would be needed to adequately power the study.

As outlined in the FDA’s draft guidance and relevant documentation concerning abiraterone acetate,^12^^,^^15^, ^16^, ^17^ a crossover study conducted under fasting conditions is advised for evaluating the bioequivalence of the test and reference products. The NMPA’s Technical Guidance for Bioequivalence Evaluation of Chemical Generic Drugs Using Pharmacokinetic Parameters^18^ specifies that if the reference drug’s instructions require administration on an empty stomach (at least 1-hour before or 2 h after meals), post-meal bioequivalence studies are unnecessary. Since the reference formulation of abiraterone acetate mandates dosing at least 1 h before or 2 h after meals, this study only evaluated bioequivalence under fasting conditions.

This study adopted a single-dose, randomized, open-label design with a three-sequence, three-period, partially replicated crossover model, in accordance with the NMPA’s bioavailability and bioequivalence study guidelines.^18^^,^^19^ A washout interval of 7 days was implemented between each dosing phase, exceeding seven times the reported elimination half-life of the drug as referenced in previous studies,^14^ the elimination half-life of abiraterone acetate in healthy subjects ranges from about 4.2 to 16.6 h, so this washout period ensured sufficient clearance between treatments.

This study adopted a three-period, three-sequence, partially replicated crossover design (TRR, RTR, RRT), in which the Reference formulation (R) was administered twice and the test formulation (T) once to each subject. This design allows direct estimation of the within-subject variance for the Reference formulation, which is essential for applying the Reference-Scaled Average Bioequivalence (RSABE) approach to highly variable drugs. Randomization was performed in equal proportions across the three sequences to ensure balanced evaluation of both formulations.

### Subjects

A total of 128 healthy Chinese male subjects were screened for this study, of which 45 participated in the study. The included subjects were aged 18-years and older, including the boundary values, with a Body Mass Index (BMI) between 19 and 26 kg/m^2^. Participants were required to weigh at least 50 kg. Before enrollment, researchers reviewed each participant’s physical exam results, medical history, and lab tests to confirm the absence of any clinically significant abnormalities. All participants provided written informed consent and were fully briefed on the study procedures prior to starting.

Exclusion criteria included a history of mental illness or substance abuse; known allergies (including drug or food allergies); excessive intake of caffeine-containing products (such as tea or coffee); alcohol consumption within the past three months; participation in another clinical trial within three months prior to screening; use of any medications (prescription, over-the-counter, traditional Chinese medicine, or supplements) within two weeks before screening; and any major illnesses or conditions that could make participation unsafe or inappropriate.

### Test procedure

Participants were admitted to the clinical center one day prior to drug administration. They were randomly allocated into three groups: TRR, RRT, and RTR. Three groups of subjects underwent a 10-hour fasting period before dosing. In the morning, both groups orally received the designated medications (T or R) with 240 mL of water. Participants were provided lunch and dinner 4 h and 10 h after drug administration. Within one hour before and after the administration, participants were only allowed to take water but no other liquids. In addition, they were advised to avoid strenuous physical activity for 4 h after taking the medication. On the 7th day, all participants returned to the trial center for further evaluation, which included interviews and vital sign checks. After fasting for 10 h, participants followed a predetermined protocol for testing and took another tablet as instructed. On day 14, the procedure was repeated, and each subject received the third dose of medication.

### Blood samples

After oral administration, the effective absorption of abiraterone and its bioequivalence were assessed by measuring drug concentrations in the blood. Venous blood samples (4 mL each) from the upper limbs were collected before and after dosing. To ensure that the sampling times cover the absorption, distribution, and elimination phases of the drug, the final time points were set at not less than three times the half-life of the drug: within 1 h before administration (0 h) and 0.25 h, 0.5 h, 1.0 h, 1.33 h, 1.67 h, 2.0 h, 2.33 h, 2.67 h, 3.0 h, 3.5 h, 4.0 h, 4.5 h, 5.0 h, 6.0 h, 8.0 h, 12.0 h, 24.0 h, 36.0 h, 48.0 h, and 72.0 h after administration. To prepare plasma for analysis, blood samples were centrifuged at 1700× g for 10-minutes at 4 °C within one hour of collection. The resulting plasma was stored at −60 °C until further analysis. An independent third-party laboratory quantified abiraterone concentrations using a validated Liquid Chromatography-tandem Mass Spectrometry (LC-MS/MS) method to minimize potential bias. A Shimadzu LC-20ADXR high-performance liquid chromatograph coupled with Shimadzu SI-30ACMP autosampler and an AB Sciex Triple Quad 4500 mass spectrometer coupled with AB Sciex Turbo Spray ion source was used for the analysis.

Although the early post-dose sampling schedule was intensive (with 11 samples collected within the first 3 h), sample collection was carried out by two trained phlebotomy teams working in parallel to minimize participant discomfort and maintain precise timing. All time points were pre-labeled and closely monitored using a digital time-tracking system, ensuring full compliance with the planned schedule. This procedure was successfully implemented for all participants without any missed or delayed samples.

### Pharmacokinetic data

Pharmacokinetic (PK) analysis was conducted using Phoenix WinNonlin (version 8.3) software (Certara, Princeton, New, USA). AUC_0-∞_, λz, and t_1/2_ from periods with AUC_%Extrap >20% were excluded from descriptive statistics due to unreliable terminal phase estimation, while these data were retained for predefined sensitivity analyses. This revision improves transparency and reproducibility. Bioequivalence between the test and reference formulations was evaluated by calculating the main pharmacokinetic parameters (C__max_, AUC_0-t_, and AUC_0-∞_) of abiraterone.^20^, ^21^, ^22^ If the individual standard deviation of a specific pharmacokinetic parameter (C__max_, AUC_0-t_, or AUC_0-∞_) in the reference formulation is <0.294, based on the mean bioequivalence method, the test formulation is considered bioequivalent to the reference formulation if the 90% Confidence Interval (CI) of the geometric mean ratio of the test formulation to the reference formulation for this parameter falls within the range of 80.00%–125.00%. The reliability of AUC_0-∞_ estimates was assessed using the percentage of extrapolated area (AUC_%Extrap). For any period in which AUC_%Extrap exceeded 20%, the corresponding AUC_0-∞_, terminal elimination rate constant (λz), and half-life (t_1/2_) were considered unreliable and were therefore excluded from descriptive statistical summaries. However, these AUC_0-∞_ values were retained and included in a predefined sensitivity analysis to assess the robustness of the bioequivalence conclusions. However, if the individual standard deviation of a specific pharmacokinetic parameter (C__max_, AUC_0-t_, or AUC_0-∞_) in the reference formulation is greater than or equal to 0.294, based on the FDA-recommended corrected mean bioequivalence method, the test formulation is considered bioequivalent to the reference formulation if the point estimate of the geometric mean ratio of the test formulation to the reference formulation for this parameter falls within the range of 80.00%–125.00%, and the critical bound value is less than or equal to 0. When the test and reference formulations are bioequivalent across all pharmacokinetic parameters (C__max_, AUC_0-t_, and AUC_0-∞_), the two formulations are considered bioequivalent. For highly variable drugs, a mixed scaling approach is used. Namely, the reference-scaled procedure is used for specific PK parameters that have a within subject variability of the reference product (sWR) ≥ 0.294.^23^ Under the Reference-Scaled Average Bioequivalence (RSABE) framework, bioequivalence is concluded when the point estimate of the geometric mean ratio falls within 80.00%–125.00% and the upper 95% confidence bound for the scaled average bioequivalence statistic (referred to as the “critical bound”) is ≤ 0.

### Safety analysis

To evaluate drug safety, participants underwent multiple assessments, including body temperature checks, physical examinations, 12-lead Electrocardiograms (ECGs), routine hematological and urine analyses, as well as blood biochemistry tests, to detect any potential Adverse Events (AEs) during the study. The severity of observed AEs was graded based on the Common Terminology Criteria for Adverse Events (CTCAE) version 5.0,^24^ and all events were coded using MedDRA terminology, categorized according to System Organ Class and Preferred Term (SOC/PT).

### Data analysis

Data are presented as the mean ± Standard Deviation (SD). Statistical analyses were performed using the SAS 9.4 software (SAS Institute, Cary, North Carolina). ANOVA was used to analyze the differences in the log-transformed primary PK parameters (C__max_, AUC_0-t_, and AUC_0-∞_). Non-parametric statistical methods were employed to test T__max_.

## Results

### Demographic data

**Fig. 1:**
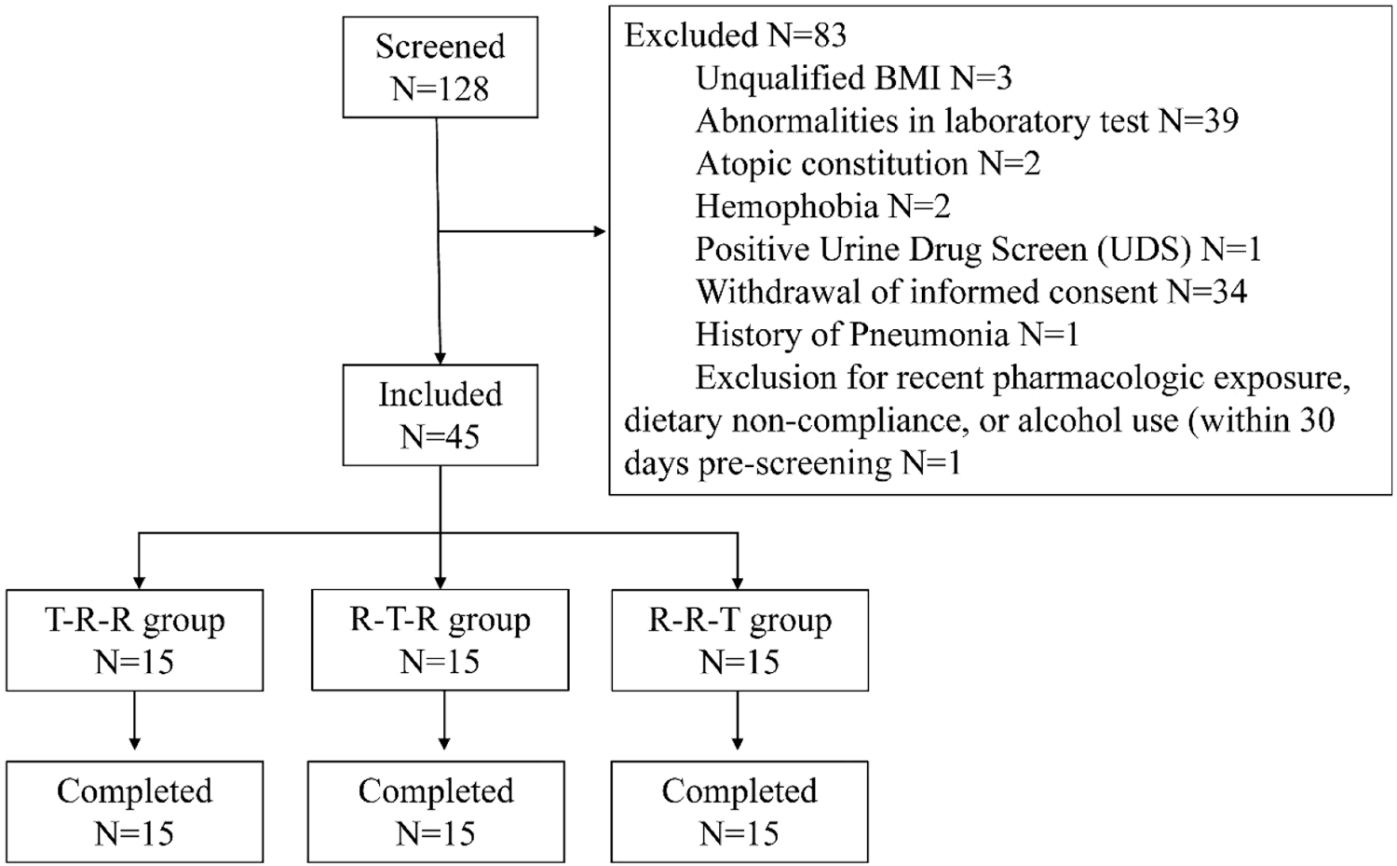
Screening procedure of healthy subjects in this study.

Preliminary screening was conducted on 128 participants according to predetermined criteria. After thorough evaluation, 45 male participants were selected for the study, and 83 participants were excluded because they did not meet the registration criteria. The screening procedure and reasons for participant exclusion are illustrated in Fig. 1. Demographic characteristics of the enrolled subjects were collected, summarized, and are displayed in Table 1.

**Table 1:** Demographic characteristics of all subjects.

Demographic	Fasting (n = 45)	Range
Age (y) mean (SD)	29.8 (5.74)	19‒40
Sex, n (%)		
Male	45 (100)	
Body weight (kg) mean (SD)	67.63 (7.383)	54.6‒80.1
Height (cm) mean (SD)	172.18 (5.785)	159‒183
BMI (kg/m^2^) mean (SD)	22.80 (2.112)	19.2‒25.6
Race, n (%)		
Han	45 (100)	

BMI, Body Mass Index; SD, Standard Deviation.

### PK analyses

**Fig. 2:**
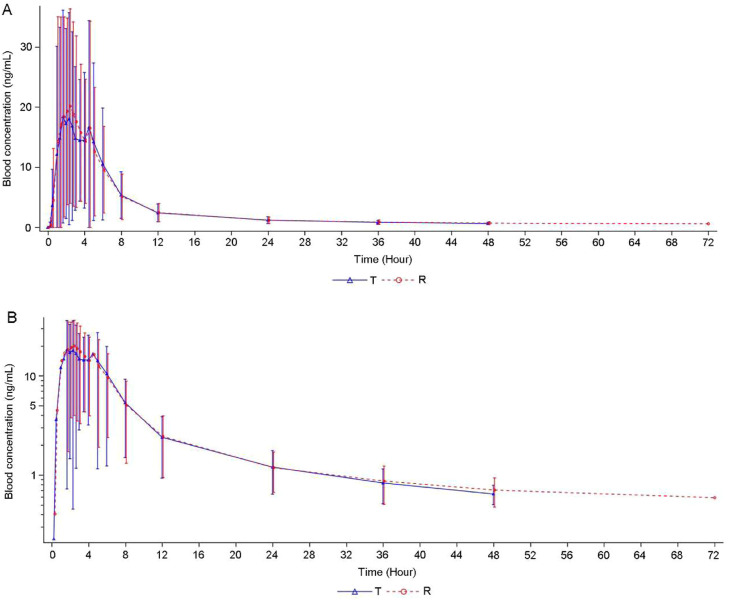
Mean (± standard error) plasma concentration-time curves of abiraterone acetate under fasting conditions. The error bars represent standard deviation. (A) Mean blood concentration-time curve of abiraterone; (B) Average plasma concentration-time semi-logarithmic curve of abiraterone.

All 45 participants completed the study successfully, and their data were included in the Pharmacokinetic Concentration Set (PKCS), Pharmacokinetic Parameter Set (PKPS), and Bioequivalence Set (BES) analyses. The mean plasma concentration-time profile of abiraterone is depicted in Fig. 2, providing an overview of the drug’s pharmacokinetics. Pharmacokinetic parameters for subjects receiving the test and reference formulations are summarized in Table 2.

**Table 2:** Primary PK evaluation (PK analysis set).

Parameter (units)	mean ± SD (%cv)	
	Test (n = 45)	Reference (n = 45)
T__max_* (h)	1.9920 (0.492, 5.992) (n = 45)	1.9920 (0.492, 5.992) (n = 90)
C__max_ (ng/mL)	31.904±22.3885 (70.17) (n = 45)	31.335±23.5214 (75.06) (n = 90)
AUC_0-t_ (h*ng/mL)	137.997±90.4209 (65.52) (n = 45)	138.813±96.8529 (69.77) (n = 90)
AUC_0-∞_ (h*ng/mL)	150.648±91.7943 (60.93) (n = 44)	150.656±99.3996 (65.98) (n = 88)
λ_z_ (1/h)	0.1208±0.0950 (78.63) (n = 44)	0.1335±0.0987 (73.93) (n = 88)
t_1/2_ (h)	9.2912±5.5894 (60.16) (n = 44)	8.5451±5.6877 (66.56) (n = 88)
AUC_%Extrap (%)	7.756±3.9793 (51.31) (n = 44)	7.648±4.2201 (55.18) (n = 88)

T__max_ is presented as median (minimum–maximum); all other parameters are presented as mean ± SD.

T__max_, Time to reach maximum concentration, shown as median (min, max); C__max_, Maximum plasma drug concentration; AUC_0-t_, Area Under the plasma Concentration-time curve from time 0 to the last measurable concentration; AUC_0-∞_, Area Under the plasma Concentration-time curve from time 0 to infinity; PK, Pharmacokinetic; T_1/2_, Elimination half-life; N, Number of cases included in PKPS and using the investigational drug. This parameter of this preparation represents the number of statistical cases of effective parameters; If the AUC_%Extrap of subjects is greater than 20%, then AUC_0-∞_, λz, t_1/2_, and AUC_%Extrap of this period are not included in the descriptive summary.

Following administration, the calculated ratios of AUC_0-t_ and AUC_0-∞_ for abiraterone were all above 80%, indicating that the 72 h blood sampling period was adequate. The elimination half-life was approximately 8h to 9 h, and since the 72-hour sampling covered more than five half-lives, it confirms the suitability of the sampling schedule. Moreover, the findings confirm that the 7-day washout period ‒ more than seven times the drug’s half-life ‒ is sufficient to fully clear the prior dose before subsequent administration, thereby reducing the risk of carryover effects.

### Bioequivalence analysis

The authors performed statistical analysis on the pharmacokinetic parameters to evaluate the bioequivalence between the test and reference drugs, as presented in Table 3. The C_max, AUC_0-t_, AUC_0-∞_, and Within-Subject variability Ratio (SWR) for abiraterone were calculated using a mixed linear model. As the SWR of the reference formulation exceeded 0.294 (CV >30%), the RSABE approach was applied in accordance with FDA guidance for highly variable drugs, which scales the BE limits to the reference variability when standard 80%–125% bounds are too restrictive. The geometric mean ratios, assessed using the RSABE method, were 104.19% (critical bound: −0.0926), 103.09% (−0.1019), and 102.74% (−0.0989), respectively. All ratios and their 90% confidence intervals fell within the predefined bioequivalence range of 80.00%–125.00%, indicating no significant differences between the test and reference drugs for these parameters.

**Table 3:** Bioequivalence analysis under fasting conditions.

Parameter (units)	Average Bioequivalence (ABE)				Reference Scaled Average Bioequivalence (RSABE)				Evaluation methodology	Conclusion
	Test (n = 45)	Reference (n = 45)	T/R (%)	90% CI (%)	S_WR_ [≥0.294]	Point Estimate [80.00, 125.00]	Critical Bound [≤0]	CVw (%)		
C__max_ (ng/mL)	25.80	24.76	104.19	[92.04, 117.95]	0.4169	104.19	−0.0926	43.57	RSABE	Bioequivalent
AUC_0-t_ (h*ng/mL)	114.53	111.10	103.09	[92.23, 115.22]	0.4269	103.09	−0.1019	44.71	RSABE	Bioequivalent
AUC_0-∞_ (h*ng/mL)	126.64	121.48	104.24	[93.51, 116.21]	0.4212	102.74	−0.0989	44.05	RSABE	Bioequivalent

When SWR ≥0.294, based on the FDA-recommended reference formulation-corrected average bioequivalence method, if the point estimate of the geometric mean ratio of the test formulation to the reference formulation for a certain pharmacokinetic parameter falls within the range of 80.00%–125.00%, and the critical bound value is less than or equal to 0, then the test and reference formulations are bioequivalent in terms of that pharmacokinetic parameter.

The test drug showed a 0.75 h delay in T__max_ compared to the reference drug; however, non-parametric tests indicated that this difference was not statistically significant (p = 0.4625 for the first reference and p = 0.0784 for the second), as shown in Table 4.

**Table 4:** Evaluation of T__max_ of abiraterone (paired non-parametric test).

Parameter	Comparable groups	*S*	p
T__max_	T VS R1	55.5000	0.4625
T__max_	T VS R2	116.5000	0.0784

For period where AUC_%Extrap > 20%, AUC_0-∞_ were excluded from the BE analysis. In case where AUC_%Extrap > 20%, the AUC_0-∞_ for the corresponding period is included in the equivalence analysis for sensitivity testing. For subjects 1033 and 1044, the first and second cycles of this trial showed an AUC_%Extrap of >20%. The results of the mixed linear model calculation indicate the following:

The SWR of AUC_0-∞_ was 0.4122, which is greater than 0.294. Therefore, the RSABE method was used for evaluation. The point estimate of the geometric mean ratio between the test and reference formulations was 103.84%, which fell within the range of 80.00%–125.00%. The critical bound value was −0.0938, which is <0. Thus, in the sensitivity analysis, the test and reference formulations were equivalent with respect to AUC_0-∞_ (Table 5).

**Table 5:** Geometric mean ratio and confidence interval of the main evaluation indicators of abiraterone ‒ sensitivity analysis.

Parameter (units)	Average Bioequivalence (ABE)				Reference Scaled Average Bioequivalence (RSABE)				Evaluation methodology	Conclusion
	Test (n = 45)	Reference (n = 45)	T/R (%)	90% CI (%)	Sw_R_ [≥0.294]	Point Estimate [80.00, 125.00]	Critical Bound [≤0]	CVw (%)		
C__max_ (ng/mL)	25.80	24.76	104.19	[92.04, 117.95]	0.4169	104.19	−0.0926	43.57	RSABE	Bioequivalent
AUC_0-t_ (h*ng/mL)	114.53	111.10	103.09	[92.23, 115.22]	0.4269	103.09	−0.1019	44.71	RSABE	Bioequivalent
AUC_0-∞_ (h*ng/mL)	125.59	120.95	103.84	[93.44, 115.39]	0.4122	103.84	−0.0938	43.04	RSABE	Bioequivalent

When SWR <0.294, based on the average bioequivalence method, if the 90% Confidence Interval of the geometric mean ratio of the test and reference formulations for a certain pharmacokinetic parameter is within the range of 80.00%–125.00%, then the test and reference formulations are considered equivalent in terms of that pharmacokinetic parameter. When SWR ≥0.294, based on the reference formulation-corrected average bioequivalence method recommended by the FDA, if the point estimate of the geometric mean ratio of the test and reference formulations for a certain pharmacokinetic parameter falls within the range of 80.00%–125.00%, and if the critical bound value is ≤0, then the test and reference formulations are considered equivalent for that pharmacokinetic parameter. In case of AUC_%Extrap of the subject >20%, the AUC0-∞ of this period is included for sensitivity analysis. A critical bound ≤0 indicates that the upper one-sided 95% Confidence bound of the RSABE test statistic is below zero and therefore satisfies the RSABE criterion.

In summary, these results indicate that the test and reference drugs are bioequivalent under fasting conditions, confirming no significant differences in the C__max_, AUC_0-t_, and AUC_0-∞_ of abiraterone. Although a delay in T__max_ was noted between the test and reference formulations, this difference does not affect the overall conclusion of their bioequivalence and merits additional study.

### Safety analysis

Throughout the trial, no serious AEs were recorded, and no participants withdrew because of adverse events. Among the 45 participants included in the safety dataset, 14 experienced 17 different AEs during the trial, with an incidence rate of 31.1%. In particular, 7 participants in the test formulation group experienced 9 different AEs, with an incidence rate of 15.6%. Similarly, 7 participants in the reference formulation group experienced 8 different AEs, with an incidence rate of 15.6%. The details of these AEs are presented in Tables 6, 7, and 8. Importantly, all AEs were mild and did not require specific treatment. Laboratory abnormalities, mainly mild and transient elevations in alanine aminotransferase, triglycerides, and total bile acids, constituted the majority of reported adverse events. These changes were asymptomatic, did not exceed clinically significant thresholds, and required no intervention, supporting the conclusion that all adverse events were mild and not clinically meaningful. All AEs were classified as of Grade 1, leading to no severe adverse reactions or participant withdrawal. Overall, these data indicate that under fasting conditions, oral abiraterone acetate tablets (250 mg) are safe and well tolerated by Chinese men. The most frequently reported treatment-emergent adverse events were laboratory abnormalities, mainly mild and transient elevations in alanine aminotransferase, blood triglycerides, and total bile acids, as well as occasional positive urinary white blood cells. These laboratory changes were asymptomatic, did not exceed clinically significant thresholds, and were not associated with clinical signs of hepatic dysfunction, metabolic disturbance, or infection. No subject exhibited alanine aminotransferase elevations greater than three times the upper limit of normal, and all abnormal values resolved spontaneously or showed a trend toward normalization without intervention. These findings support the conclusion that all adverse events were mild and not clinically meaningful.

**Table 6:** Summary of Treatment-Emergent Adverse Events (TEAEs) (safety analysis set).

	Test^a^ (n = 45)		Reference^b^ (n = 45)		Total^c^ (n = 45)	
	n (%)	Events	n (%)	Events	n (%)	Events
**AE**	7(15.6%)	9	7 (15.6%)	8	14 (31.1%)	17
**ADR**	7 (15.6%)	9	7 (15.6%)	8	14 (31.1%)	17
**SAE**	0	0	0	0	0	0
**SAR**	0	0	0	0	0	0
**AE leading to withdrawal**	0	0	0	0	0	0
**ADR leading to withdrawal**	0	0	0	0	0	0
**Grades**						
Grade 1 Mild	7 (15.6%)	9	7 (15.6%)	8	14 (31.1%)	17
Grade 2 Moderate	0	0	0	0	0	0
Grade 3 Severe	0	0	0	0	0	0
Grade 4 Life-threatening consequences	0	0	0	0	0	0
Grade 5 Death	0	0	0	0	0	0
**Ending**						
Disappearance, with aftereffects	‒	0	‒	0	‒	0
Disappear without any aftereffects	‒	4	‒	3	‒	7
Relief	‒	0	‒	0	‒	0
Continue	‒	0	‒	0	‒	0
Serious	‒	0	‒	0	‒	0
Lost to follow-up	‒	5	‒	5	‒	10
Death	‒	0	‒	0	‒	0
Other	‒	0	‒	0	‒	0
**Correlation**						
Definitely	0	0	0	0	0	0
Probably	0	0	0	0	0	0
Possibly	7 (15.6%)	9	7 (15.6%)	8	14 (31.1%)	17
Unlikely	0	0	0	0	0	0
Not Related	0	0	0	0	0	0

Adverse Drug Reaction (ADR) is defined as an AE that is definitely, probably, or possibly related to the drug. According to the principle of conservatism, when the relationship between an AE and the drug is missing, the AE is counted as an ADR. In this partially replicated crossover study, safety summaries are presented by number of subjects, not by number of dosing exposures. Although each subject received two doses of the reference formulation, n = 45 indicates the number of subjects who received at least one dose of the reference formulation.

N, Number of subjects in the Safety Population.

a Subjects who received test formulation.

b Subjects who received at least one reference formulation.

c Subjects who received at least one study treatment.

**Table 7:** Adverse events based on the System Organ Classification (SOC) (SS).

System Organ Class	Test (n = 45)		Reference (n = 45)		Total (n = 45)	
Preferred Term	n (%)	Events	n (%)	Events	n (%)	Events
At least one adverse event	7 (15.6%)	9	7 (15.6%)	8	14 (31.1%)	17
Investigations	7 (15.6%)	9	7 (15.6%)	7	14 (31.1%)	16
Alanine aminotransferase increased	3 (6.7%)	3	1 (2.2%)	1	4 (8.9%)	4
White blood cells urine positive	0	0	2 (4.4%)	2	2 (4.4%)	2
Blood bilirubin increased	1 (2.2%)	1	0	0	1 (2.2%)	1
Blood triglycerides increased	4 (8.9%)	4	3 (6.7%)	3	7 (15.6%)	7
Testosterone decreased	0	0	1 (2.2%)	1	1 (2.2%)	1
Total bile acid increased	1 (2.2%)	1	0	0	1 (2.2%)	1
Vascular disorders	0	0	1 (2.2%)	1	1 (2.2%)	1
Hypotension	0	0	1 (2.2%)	1	1 (2.2%)	1

For number of subjects (n) and percentage (%), if >1 AE occurs with the same preferred term (or system organ class) for the same subjects, then the subjects will be counted only once for that preferred term (or system organ class).

In this partially replicated crossover study, safety summaries are presented by number of subjects, not by number of dosing exposures. Although each subject received two doses of the reference formulation, n = 45 indicates the number of subjects who received at least one dose of the reference formulation.

**Table 8:** Adverse drug reaction based on the System Organ Classification (SOC) (SS).

System Organ Class	Test (n = 45)		Reference (n = 45)		Total (n = 45)	
Preferred Term	n (%)	Events	n (%)	Events	n (%)	Events
At least one treatment related AE	7 (15.6%)	9	7 (15.6%)	8	14 (31.1%)	17
Investigations	7 (15.6%)	9	7 (15.6%)	7	14 (31.1%)	16
Alanine aminotransferase increased	3 (6.7%)	3	1 (2.2%)	1	4 (8.9%)	4
White blood cells urine positive	0	0	2 (4.4%)	2	2 (4.4%)	2
Blood bilirubin increased	1 (2.2%)	1	0	0	1 (2.2%)	1
Blood triglycerides increased	4 (8.9%)	4	3 (6.7%)	3	7 (15.6%)	7
Testosterone decreased	0	0	1 (2.2%)	1	1 (2.2%)	1
Total bile acid increased	1 (2.2%)	1	0	0	1 (2.2%)	1
Vascular disorders	0	0	1 (2.2%)	1	1 (2.2%)	1
Hypotension	0	0	1 (2.2%)	1	1 (2.2%)	1

For number of subjects (n) and percentage (%), if >1 AR occurs with the same preferred term (or system organ class) for the same subjects, then the subjects will be counted only once for that preferred term (or system organ class). In this partially replicated crossover study, safety summaries are presented by number of subjects, not by number of dosing exposures. Although each subject received two doses of the reference formulation, n = 45 indicates the number of subjects who received at least one dose of the reference formulation.

## Discussion

Prostate cancer represents a significant health concern worldwide among men,^25^ with its incidence rising in recent years. While the precise etiology is still not fully understood, many studies indicate associations with factors such as age, ethnicity, genetic predisposition, sexual behavior, and diet. An imbalance in androgen levels within the body increases the risk of developing prostate cancer.^26^ The early symptoms of prostate cancer are often subtle and include frequent urination, urgency, incomplete voiding, and difficulty in urinating. Late-stage prostate cancer has more pronounced symptoms, such as difficulty in urinating, blood in the urine, frequent urination, urgency, and increased nocturia. If bone metastasis occurs, patients may experience pain in the lower back and legs, and in severe cases, paralysis.^27^^,^^28^

The pharmacokinetic parameters observed in the present study are consistent with those reported in previous bioequivalence studies of abiraterone acetate conducted in Chinese healthy volunteers. For example, Wang et al. reported a mean C__max_ of approximately 30–35 ng/mL, AUC_0-t_ of 120–150 h/ng/mL, and terminal half-life ranging from 7 to 12 h under fasting conditions.^14^ In our study, the mean C__max_ (∼32 ng/mL), AUC_0-t_ (∼138h/ng/mL), and t_1/2_ (∼8–9 h) fall well within these previously reported ranges, supporting the external validity of our pharmacokinetic data and confirming that the study population and analytical methodology are comparable to prior investigations.

Prostate cancer growth primarily depends on androgen-mediated signaling. Androgen deprivation therapy can reduce androgen levels using either drug or surgical castration, thereby reducing androgen receptor signaling and leading to tumor regression. However, the effects of androgen deprivation therapy are not long lasting, and eventually, all patients develop metastatic castration-resistant prostate cancer. Abiraterone, an oral inhibitor of androgen biosynthesis, inhibits the synthesis of 17α-hydroxylase, which in turn inhibits the progression of prostate cancer.^29^^,^^30^ Based on these findings, abiraterone was developed and is currently used in clinical practice.

In response to the increasing incidence of prostate cancer and the demand for affordable treatments, the authors carried out a clinical trial to assess the bioequivalence between a generic abiraterone formulation and its brand-name version. This study aimed to validate the clinical applicability of the generic product. Our findings indicated no significant difference in C__max_ values between the test and reference formulations. Based on concentration–time profiles, the authors calculated AUC_0-t_ and AUC_0-∞_, which like C__max_ data, also did not differ significantly between the two formulations. These findings indicated that the absorption profiles of the generic and branded abiraterone are comparable in healthy subjects.

Although the 0.75-hour delay in T__max_ observed for the test formulation was not statistically significant, this difference may carry pharmaceutical implications. Variations in excipient composition, tablet hardness, granule size, or coating properties between the test and reference formulations could slightly influence the dissolution rate and subsequent absorption profile of abiraterone acetate. Given that abiraterone is a poorly soluble drug with known dissolution-rate-limited absorption, minor formulation differences can transiently affect the onset of systemic exposure. However, since both the rate (C__max_) and extent (AUC_0-t_ and AUC_0-∞_) of absorption met the bioequivalence criteria, the observed delay is unlikely to impact clinical efficacy or safety. Nonetheless, these findings underscore the importance of controlling critical quality attributes ‒ particularly dissolution behavior ‒ when developing generic formulations of highly variable compounds such as abiraterone acetate.

Abiraterone acetate is a poorly water-soluble compound whose absorption is known to be dissolution-rate limited. Small differences in excipient composition, particle size distribution, solid-state properties, or tablet compression force can alter wetting, disintegration, and dissolution behavior, thereby modestly shifting the onset of absorption without affecting overall exposure. Previous formulation studies of poorly soluble drugs have demonstrated that changes in surfactants, disintegrants, or polymeric binders may delay or accelerate T__max_ while leaving C__max_ and AUC unchanged. Therefore, the observed 0.75 hour delay in T__max_ for the test formulation is most likely attributable to minor formulation-dependent differences in early dissolution kinetics rather than a true difference in bioavailability. Because both the rate (C__max_) and extent (AUC) of absorption met bioequivalence criteria, this slight temporal shift is unlikely to have clinical relevance.

Two adverse events of particular pharmacological interest were decreased testosterone levels and mild hypotension. Abiraterone acetate inhibits CYP17-mediated androgen biosynthesis, leading to suppression of testosterone production; therefore, a decrease in circulating testosterone represents an expected pharmacodynamic effect rather than an adverse safety signal. Similarly, inhibition of adrenal steroid synthesis may secondarily affect mineralocorticoid balance, which has been associated with blood pressure changes in some individuals. In the present study, the observed decrease in testosterone and single case of mild hypotension were transient, asymptomatic, and did not require intervention. These findings are consistent with the known mechanism of action and established safety profile of abiraterone acetate, further supporting that no clinically meaningful safety concerns were identified.

Furthermore, all observed AEs were classified as of grade 1 and assessed as mild according to the General Terms for Adverse Events. They resolved during the treatment period; none were considered serious. These findings indicate that the test formulation is safe and could serve as a potential alternative to the reference formulation in Chinese patients with prostate cancer.

## Conclusion

Under fasting conditions, the test and reference 250 mg abiraterone acetate tablets demonstrated bioequivalence in healthy Chinese volunteers, displaying comparable pharmacokinetic profiles. Additionally, safety assessments during the trial confirmed that both the generic and branded formulations were well tolerated and safe in the study population. These findings strongly support considering generic drugs as effective alternatives for treating prostate cancer.

## Data availability

Data will be made available on reasonable request.

## Authors’ contributions

Jinjin Shi, Xiaoshan Zhu, and Tiandong Zhang designed experiments. Xu Zuo, Ancheng Kuang and Yang Wang carried out experiments. Cheng Liu and Guiying Chen analyzed experimental results. Jinjin Shi and Xiaoshan Zhu wrote the manuscript. Tiandong Zhang revised the manuscript. All authors approved the final manuscript.

## Funding

This study was supported by the Henan Province Medical Science and Technology Tackling Program Joint Co-Construction Project (No. LHGJ20221004) and the Henan Province Medical Science and Technology Tackling Program Joint Co-Construction Project (No. LHGJ20191332).

## Declaration of competing interest

The authors declare no conflicts of interest.
